# Omics Profiles of Non-GM Tubers from Transgrafted Potato with a GM Scion

**DOI:** 10.14252/foodsafetyfscj.D-22-00010

**Published:** 2023-02-11

**Authors:** Taira Miyahara, Takumi Nishiuchi, Nao Fujikawa, Taichi Oguchi, Akira Kikuchi, Ken-ichiro Taoka, Takumi Ogawa, Karuna Honda, Yube Yamaguchi, Tomofumi Mochizuki, Daisaku Ohta, Hiroaki Kodama

**Affiliations:** 1 Graduate School of Horticulture, Chiba University, 1-33 Yayoi-cho, Inage-ku, Chiba 263-8522, Japan; 2 Division of Life Science, Graduate School of Natural Science and Technology, Kanazawa University, Kakuma, Kanazawa, Ishikawa 920-1192, Japan; 3 Division of Integrated Omics Research, Bioscience Core Facility, Research Center for Experimental Modeling of Human Disease, Kanazawa University, 13-1 Takaramachi, Kanazawa, Ishikawa 920-8640, Japan; 4 Graduate School of Life and Environmental Sciences, University of Tsukuba, 1-1-1 Tennodai, Tsukuba, Ibaraki 305-8572, Japan; 5 Faculty of Life and Environmental Sciences, University of Tsukuba, 1-1-1 Tennodai, Tsukuba, Ibaraki 305-8572, Japan; 6 Tsukuba Plant Innovation Research Center, University of Tsukuba, 1-1-1 Tennodai, Tsukuba, Ibaraki 305-8572, Japan; 7 Kihara Institute for Biological Research, Yokohama City University, 641-12 Maioka-cho, Totsuka-ku, Yokohama, 244-0813, Japan; 8 Institute of Life and Environmental Sciences, University of Tsukuba, 1-1-1 Tennodai, Tsukuba, Ibaraki 305-8572, Japan

**Keywords:** FLOWERING LOCUS T protein, genetically modified plants, grafting, new plant breeding technology, omics analysis, steroidal glycoalkaloids

## Abstract

“Transgrafting” is a grafting procedure whereby a transgenic plant body is grafted to a non-transgenic plant body. It is a novel plant breeding technology that allows non-transgenic plants to obtain benefits usually conferred to transgenic plants. Many plants regulate flowering by perceiving the day-length cycle via expression of *FLOWERING LOCUS T (FT)* in the leaves. The resulting FT protein is translocated to the shoot apical meristem via the phloem. In potato plants, FT is involved in the promotion of tuber formation. Here we investigated the effects of a genetically modified (GM) scion on the edible parts of the non-GM rootstock by using potato plants transformed with *StSP6A*, a novel potato homolog of the *FT* gene. Scions prepared from GM or control (wild-type) potato plants were grafted to non-GM potato rootstocks; these were designated as TN and NN plants, respectively. After tuber harvest, we observed no significant differences in potato yield between TN and NN plants. Transcriptomic analysis revealed that only one gene—with unknown function—was differentially expressed between TN and NN plants. Subsequent proteomic analysis indicated that several members of protease inhibitor families, known as anti-nutritional factors in potato, were slightly more abundant in TN plants. Metabolomic analysis revealed a slight increase in metabolite abundance in NN plants, but we observed no difference in the accumulation of steroid glycoalkaloids, toxic metabolites found in potato. Finally, we found that TN and NN plants did not differ in nutrient composition. Taken together, these results indicate that *FT* expression in scions had a limited effect on the metabolism of non-transgenic potato tubers.

## 1. Introduction

Conventional plant breeding involves cross-pollination and selection of progeny plants. In the late 1990s, *Agrobacterium*-mediated transformation enabled the production of plants with novel traits that improved agricultural productivity via increasing yield, reducing pesticide use, and/or modifying nutrient composition. Because genetically modified plants (GM plants) contain foreign DNA, they are regulated by many governments, and developers of GM plants must therefore prepare large amounts of data for safety assessment of GM plants in view of foods, feeds, and environments^1^^)^. In contrast, plants produced by new plant breeding technologies (NPBTs) contain no additional foreign DNA in their edible parts. Agricultural productivity can be improved efficiently by NPBTs since the associated regulatory burden is significantly lower than it is for GM-based plant breeding programs. Novel technologies including genome editing, cisgenesis and intragenesis, and RNA-directed DNA methylation (RdDM), among others, are recognized as NPBTs^2^^,^^3^^)^. Genome editing technologies enable direct editing of the genome sequence at targeted sites, and are realized by site-directed DNA nuclease technology and oligonucleotide-directed mutagenesis^3^^)^. Genome editing has the potential to significantly improve the agricultural traits of crops without the involvement of foreign DNA, and several companies have already commercialized varieties of genome-edited crops^3^^,^^4^^)^.

Grafting is a traditional technology that permits improvement of agricultural productivity in commercially valuable cultivars^5^^)^. Grafting can be used for numerous applications—for example, size reduction is widely used in apple and other fruit trees. Planting apple trees grafted on dwarfing rootstocks enables plantations to realize higher tree densities and, consequently, a higher number of fruit per area under cultivation^5^^,^^6^^)^. Another application of grafting involves improving stress tolerance, which can be imparted from a stress-tolerant rootstock to a stress-sensitive scion. A notable example is grafted grapevines. European grapes that are sensitive to phylloxera, an insect pest of grapes, can be grafted onto phylloxera-resistant rootstock^7^^)^. In addition, most citrus trees are cultivated as grafted trees to improve their stress tolerance, and genetic transformations of the citrus rootstock have been used to improve traits^8^^)^.

Grafting a non-GM scion onto a GM rootstock (“transgrafting”) is also considered to be an NPBT, since the resulting fruits on the scion do not contain foreign DNA^2^^,^^9^^)^. Moreover, most transgrafting experiments do not cause food safety concerns related to edible plant parts. Small chemical substances, RNA, and proteins have been found to move from rootstock to scion (and vice versa) via vascular tissue^9^^,^^10^^)^. Therefore, the nature of any health risks related to the edible parts of transgrafted plants should be investigated prior to commercialization, even if fruits produced on a non-GM scion are expected to be handled as non-GM foods. An earlier experiment used omics analyses to assess the effects of a GM tomato rootstock producing a transgenic protein (β-glucuronidase) in non-GM fruits. These results suggested that transgrafting did not induce genetic or metabolic variation in the scion^11^^)^. Next, in a previous paper we produced transgrafted tobacco plants using an RdDM-inducing rootstock^12^^)^. In this study, we evaluated the transcriptomic, proteomic, and metabolic effects of long-distance movement of small RNAs produced at a transgenic locus within a GM rootstock grafted to a non-GM scion. These results showed that grafting onto an RdDM rootstock caused slight changes in the transcriptomic and metabolic profiles of the non-GM scion. However, we could not find a causal relationship between the siRNAs introduced by the rootstock and these slight transcriptomic and metabolomic changes^12^^)^. Taken together, these reports show that there is no apparent food safety risk associated with the transgrafting of GM rootstocks.

In the field, grafting has not been commercially developed for the common potato (*Solanum tuberosum*). However, in laboratory experiments tomato scions have been grafted onto potato rootstocks, thereby creating plants with two kinds of sink organs in a single chimeric plant body, namely tomato fruits and potato tubers^13^^,^^14^^)^. Recent reports of potato transgrafting experiments present new strategies for the modulation of gene expression in grafted potato tubers^15^^)^. The main method of potato cultivation is vegetative propagation by tuber. Epigenetic variation (e.g., DNA methylation), is therefore stably maintained in propagated progeny tubers, and DNA methylation patterns in these tubers can be controlled by GM scions showing RdDM^15^^)^. Because DNA methylation induced by transgrafting is maintained during vegetative propagation of progeny tubers, the epigenetic modification of potato plants may become one a key NPBT application. With respect to food safety, potato constitutively synthesizes steroidal glycoalkaloids (SGAs) as major secondary metabolites. SGAs, especially α-solanine, are known to be harmful to humans, and cause gastrointestinal symptoms after SGA consumption of >1.0 mg/kg body weight^16^^)^. Interestingly, SGA levels often vary among transgenic potatoes even when transgene functions are independent of the SGA synthesis pathway^17^^,^^18^^)^. Here we investigated the effects of a GM potato scion on the traits of non-GM tubers. As a model case of the grafted potato, we transformed commercial potato plants using a transgene encoding the mobile flowering signal, florigen. FLOWERING LOCUS T (FT) is a major protein in the florigen complex^19^^)^ of *Arabidopsis thaliana*, and we used potato plants transformed with a potato homolog of FT, *StSP6A*^20^^)^. Scions transformed with the *StSP6A* gene were then grafted onto wild-type potato rootstocks. After cultivation, the tubers harvested from the rootstock were subjected to multi-omics analyses. Finally, we also examined nutrient and SGA levels to provide data for food safety assessment.

## 2. Materials and Methods

### 2.1 Grafted Potato Plants Consisting of a transgenic Scion and a non-transgenic Rootstock

Transgenic potatoes (*Solanum tuberosum* L. cultivar “Sayaka”) expressing the *StSP6A* gene, which codes for a homolog of FT found in potato and is involved in tuberization, under the control of a cauliflower mosaic virus (CaMV) 35S promoter generated by Agrobacterium-mediated transformation with pGWB5 were generated by a previous study^20^^)^. *StSP6A* ox (35:StSP6A #7) transgenic plants were used throughout this study. Transgenic and non-transgenic potato plants were cultured for five weeks in a culture room at 25°C on a 16 h (fluorescent) light/8 h dark photoperiod after which they acted as donors for grafted plants. The transgenic potato plantlets were used as scions and were grafted onto non-transgenic potato root stocks (TN). After habituation in a cultivation room, grafted potato plants were transferred into 30-cm diameter pots in netted screenhouse. Tubers were harvested after three months of cultivation. Harvested tubers were then stored for approximately one month in a cultivation room in the dark. Tubers were then cut into ~2 mm thick slices and flash-frozen in liquid nitrogen. The outermost and innermost tuber slices were used for transcriptome analysis and all other analyses, respectively. Grafted plants included non-transgenic potato rootstocks grafted with a non-transgenic scion (NN), which was used as a control.

### 2.2 Transcriptomic Analysis of Grafted Potato Plant Tubers

Total RNA was extracted from potato tuber samples (including the skin), using a Favoprep Plant Total RNA Mini Kit (Favogen Biotech Crop., Taiwan). We outsourced RNA library construction and mRNA sequencing to Eurofins Genomics (Tokyo, Japan). mRNA was purified as poly(A)+ RNA, and paired-end 150bp sequencing data was generated using a NovaSeq 6000 platform (Illumina, San Diego, USA). The resulting mRNA-seq dataset (BioProject ID: PRJDB13103, RUN ID: DRR350765-70) contained a total of 447.6 million reads. Adapter sequences were then trimmed, and low-quality reads containing poly-N sequences and/or that were than 50 bp in length were discarded using fastp (version 0.20.1). After trimming, read data were aligned to the transcriptome dataset (DM_1-3_516_R44_potato.v6.1.hc_gene_models.cdna.fa) registered at potato genome database (Spud DB: http://spuddb.uga.edu/index.shtml) using Bowtie2 version 2.4.4. RSEM (version 1.3.3) was used to calculate gene expression levels, which were expressed as transcripts per million (TPM). Principal component analysis and the identification of differentially expressed genes (DEGs) were performed using edgeR (version 3.34.1), and R (version 4.1.0).

### 2.3 Proteomic Analysis of Grafted Potato Plant Tubers

For each 1g of potato tuber tissue, we added 10 g of 7 M urea and mashed the resulting mixture in a mortar and pestle. The homogenate was then placed in a tube and kept at room temperature for 30 minutes. This was then sonicated four times at one-second intervals before being centrifuged at 1,300 rpm for 30 minutes at 20°C. Next, 250 µL of the centrifuged supernatant was transferred to a new tube, to which three times its volume in acetone chilled to −20°C was added. After vortexing, tubes were stored at −20°C overnight. Two centrifugations were then performed to completely remove the acetone; each centrifugation was 15,000 rpm for 15 minutes at 4°C. Tubes were then air-dried for five minutes. Finally, 30 µL of 7M urea was added to completely dissolve the precipitate, and the protein content was quantified using a Qubit Protein Assay Kit (Thermo Fisher Scientific: Massachusetts, US). To conduct further analyses, all protein samples (10 µg) were adjusted to a final volume of 10 µL in 6 M Urea and 50 mM triethylammonium bicarbonate (TEAB, pH 8.5). These proteins were then reduced, alkylated, and digested by trypsin. The trypsin-digested peptides were purified and separated using a liquid chromatography (LC) system (EASY-nLC 1200; Thermo Fisher Scientific, Waltham, USA). The peptide ions were detected using mass spectroscopy (MS; Orbitrap QE plus MS; Thermo Fisher Scientific). MS/MS searches were then carried out by using SEQUEST HT search algorithms implemented in Proteome Discoverer (PD) 2.2 (Version 2.2.0.388; Thermo Fisher Scientific) to find query sequences in the DM_1-3_516_R44_potato.v6.1.hc_gene_models.pep.fa file, which is registered on the Spud DB potato genome database site. Label-free quantification was also performed with PD 2.2 using precursor ion quantifier nodes. Abundance normalization was performed using the total peptide amount mode. The biological processes and molecular functions of the proteins whose accumulations were found to differ between the two groups were further investigated using the DAVID Bioinformatics Resource (https://david.ncifcrf.gov/tools.jsp, 2021 update).

### 2.4 Metabolomic Analysis of Potato Root Stock Tubers

For metabolomic analyses, we used potato tubers obtained from six independent grafted potato plants (i.e., three NN lines and three TN lines). These tubers were subjected to metabolomic analysis using LC-ESI-MS and GC-EI-MS.

#### 2.4.1 LC-ESI-MS analysis of hydrophilic compounds

Untargeted metabolomic analyses of potato tuber samples were conducted using a Q Exactive orbitrap mass spectrometer (Thermo Fisher Scientific) connected to an UltiMate 3000 Rapid Separation LC system (Thermo Fisher Scientific). Metabolites in potato tuber samples were extracted using a methanol:water (4:1, v/v) mixture. Highly hydrophobic compounds in the extracts were removed using a MonoSpin C18 column (GL Sciences Inc., Tokyo, Japan) pre-equilibrated with a methanol:water (4:1, v/v) mixture. After filtration using a 0.2 μm pore size membrane filter, filtrate samples (2 μl) were then subjected to LC-MS analysis. Chromatographic separation was carried out in an InterSustain AQ-C18 column (2.1 x 150 mm, 3 μm particle size, GL Sciences Inc.). The mobile phase was 0.1% (v/v) formic acid in water (A) and acetonitrile (B). The gradient program was set as follows: 2% B, constant for 3 min, followed by an increase to 98% B over 30 min, constant flow for 5 min, and finally a reduction to 2% B over 0.1 min. The post-run equilibrium time was 4.9 min. The flow rate was kept constant at 0.2 ml min^−1^. The column oven was kept at 40°C. Mass spectrometry was performed using a Q Exactive mass spectrometer that was set to operate in the ESI positive ion mode. All spectra were acquired in the range of *m*/*z* 80−1200. Tandem mass spectrometry was performed by collision-induced dissociation for the ten most intense ions of the full mass scan. Dynamic exclusion was set at 20 sec. The raw data file was then converted to the mzXML file format using ProteoWizard’s MSConvertGUI software (http://proteowizard.sourceforge.net). PowerGetBatch software^21^^)^ was used for peak detection, characterization, and alignment. Finally, peaks were annotated using the UC2 database^22^^)^ and the MFSearcher program^23^^)^ with the predicted mass values of the original molecules at 5 ppm mass tolerance together with the KNApSAcK^24^^)^ and HMDB^25^^)^ database records.

#### 2.4.2 LC-ESI-MS analysis of hydrophobic compounds

Lipidome analyses of potato tuber samples were conducted using a Q Exactive mass spectrometer (Thermo Fisher Scientific) connected to an UltiMate 3000 Rapid Separation LC system (Thermo Fisher Scientific). The metabolites present in potato tuber samples were extracted with a methanol: methyl *tert*-butyl ether (3:10, v/v) mixture. After adding 1.25 volumes of water to each extract, the resulting mixture was centrifuged at 3,000 x *g* for 10 min. The methyl *tert*-butyl ether phase was then collected and filtered through a membrane filter with pores 0.2 μm in size. The filtrate (2 μl) was then subjected to LC-MS analysis. Chromatographic separation was carried out using a SunShell C18 column (2.1 x 150 mm, 2.6 μm particle size, ChromaNik Technologies Inc., Osaka, Japan). The mobile phase contained 0.1% (v/v) formic acid and 10 mM ammonium formate in an acetonitrile: water (60:40, v/v) mixture (A) and 0.1% (v/v) formic acid and 10 mM ammonium formate in a 2-propanol:acetonitrile (90:10, v/v) mixture (B). The gradient program was set as follows: 30% B to 35% B within 10 min, 35% B to 55% B within 10 min, 55% B to 65% B within 5 min, 65% B to 100% B within 10 min, constant flow for 10 min, and finally a reduction of B to 30% in 0.1 min. The post-run equilibrium time was 4.9 min, and the flow rate was kept constant at 0.2 ml min^−1^. The column oven was maintained at 40°C. Next, mass spectrometry was performed on a Q Exactive mass spectrometer. This instrument was set to operate in the ESI with positive/negative ion polarity switching. All spectra were acquired in the range of *m*/*z* 80−1200. Tandem mass spectrometry was performed by collision-induced dissociation for the five most intense ions of the full mass scan. Dynamic exclusion was set at 20 sec. Once again, the raw data file was converted to mzXML by MSConvertGUI. PowerGetBatch^21^^)^ and Lipid Search^26^^)^ were used for all data analyses. The identification of lipids was performed based on MS/MS spectrum matching between the original molecules and querying of the Lipid Search database by the product search function of Lipid Search.

#### 2.4.3 GC-EI-MS analysis of polar and nonpolar compounds

Metabolite extraction, phase separation, and derivatization for GC-EI-MS analysis of potato tuber samples were performed according to the method of Ogawa et al (2017)^27^^)^. Briefly, metabolites were extracted from 30 mg samples of ground and lyophilized potato tubers using 650 µL of extraction solvent [methanol/chloroform/H_2_O (3:1:1, v:v:v)]. Ribitol (100 µg/mL) and testosterone (10 µg/mL) were present as internal controls. The extraction procedure was repeated twice, and the extracts were combined into a single tube. Next, 800 µL of extract was used for phase separation. 50 µL of the polar fraction was dried, then subjected to oximation and trimethylsilylation. The whole nonpolar fraction was dried and subjected to oximation and trimethylsilylation after fatty acid esterification. Next, we analyzed 1µL of derivatized sample by GC-EI-MS on an Agilent 6890 Gas Chromatograph (Agilent Technologies Inc., Santa Clara, USA) and a Micromass GCT Premier Mass Spectrometer (Waters Co., Milford, USA). The GC was equipped with an Agilent HP-5 ms column, which had a 30 m × 0.25-mm inner diameter and a film thickness of 0.25µm (Agilent Technologies Inc.). Analytical conditions were same as those reported by Ogawa et al (2017)^27^^)^. MetAlign^28^^)^ was used for ion extraction from the total ion current (TIC) chromatograms obtained by GC-EI-MS and for peak alignment between samples. AIoutput2^29^^)^ was used for peak deconvolution and automatic peak identification. An in-house mass spectral library was used for peak annotation.

### 2.5 Nutrient Composition Analysis

Analyses of nutrient composition (i.e., the relative abundance of water, proteins, lipids, ash, and carbohydrates) were conducted by Japan Food Research Laboratories (Tokyo, Japan). These analyses were performed using frozen potato slices approximately 30 g in mass.

### 2.6 Statistical Analysis

Principal component analysis (PCA) and Student t-tests were performed for metabolomic analysis using MetaboAnalyst version 5.0^30^^)^. Auto scaling was selected as the data standardization method for PCA. Tukey HSD (honestly significant difference) tests were performed using R version 4.1.3 (2022-03-10) (The R Foundation for Statistical Computing, Vienna, Austria).

## 3. Results

### 3.1 Plant and Tuber Traits of Non-transgenic Potato Rootstocks Scioned with a transgenic Potato Scion

**Fig. 1.**
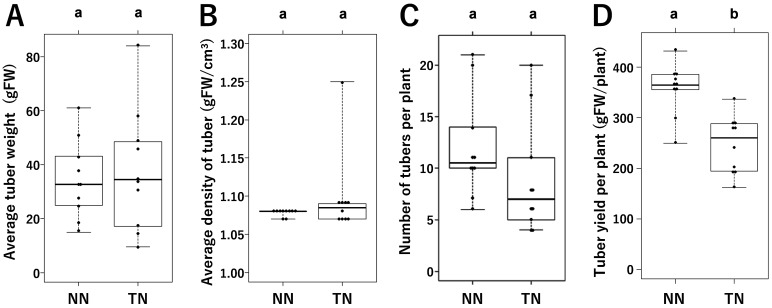
Properties of tubers and tuber productions of grafted plants Panels A and B show average tuber weights and densities for non-transgenic potato rootstocks scioned with non-transgenic scions (NN) or transgenic potato scions (TN). Panels C and D show the number of tubers and the tuber yield per individual for NN and TN-grafted potato plants, respectively. Different letters above the bars indicate significant differences among grafted plants as determined by Tukey HSD test (ɑ=0.05). Error bars indicate standard error.

Ten grafted plants of each type—i.e., non-transgenic rootstocks scioned with a transgenic scion (TN) and non-transgenic rootstocks scioned with a non-transgenic scion (NN)—were cultivated for approximately three months from late January 2020. Cultivation took place in pots inside a net house. Tubers were harvested from late April to early May. We found no significant differences between TN- and NN-grafted plants with respect to tuber number, average tuber weight, or specific gravity, but we did find statistically significant differences in total tuber yield per plant (**Fig. 1**). We selected three TN- and four NN-grafted plants for subsequent analysis of their tubers; this selection was performed to obtain plants that formed tubers of relatively uniform size with less green skin discoloration of the skin.

### 3.2 Transcriptomic Analysis of Tubers from NN- and TN-grafted Potato Plants

**Fig. 2.**
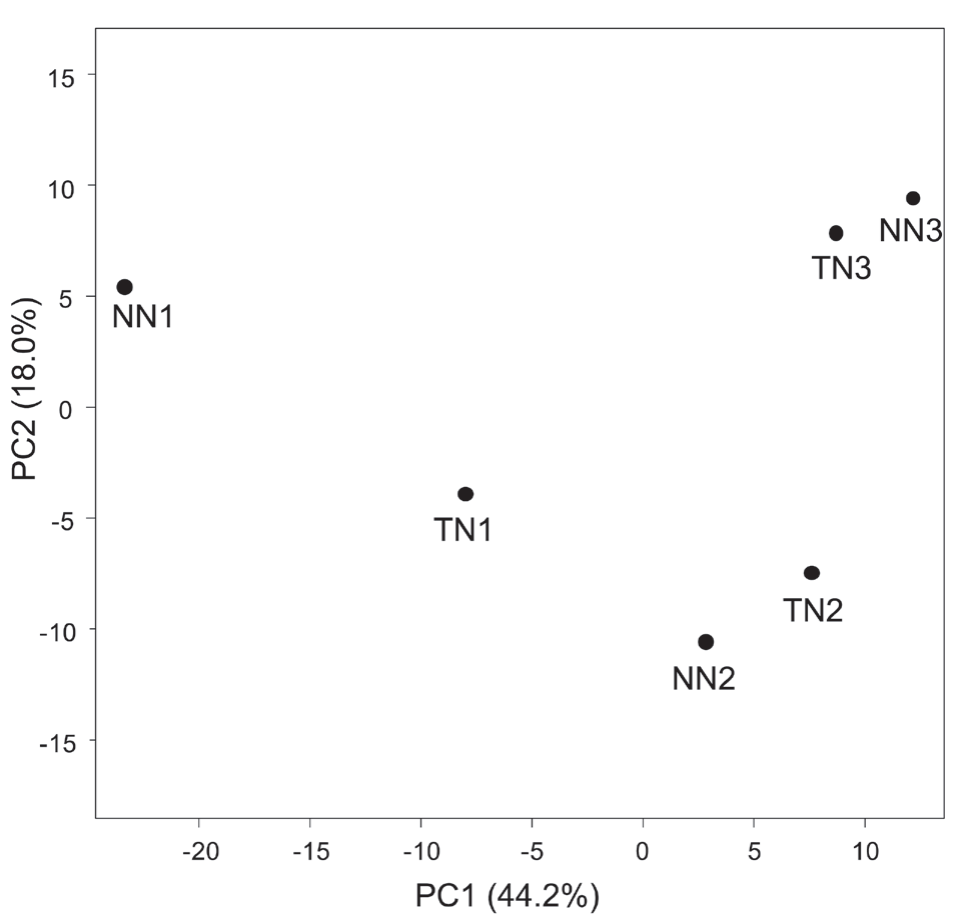
Principal component analysis of transcriptome data for the NN and TN lines. Each plot represents an individual sample of three biological replicates. Percentage values in parentheses indicate the respective contribution ratios.

Alignment of our transcriptomic data to the potato cDNA sequence template (DM_1-3_516_R44_potato.v6.1.hc_gene_models.cdna.fa) showed that approximately 70% of read data were aligned for each sample. The number of genes whose expression was confirmed in at least one sample was 35,823. PCA of the expression data for these genes identified no clusters in the NN and TN lines (**Fig. 2**). Therefore, we did not observe a clear difference in gene expression between the TN and NN lines. We identified only one DEG, which was up-regulated in the NN line compared to the TN line. The function of this gene (Soltu.DM.01G027590.1) has not yet been identified. The expression of this gene was detected in NN1 (TPM: 19.41) and NN3 (TPM: 23.31), but it was almost absent in NN2 (TPM: 0.78), as well as in all replicates from the TN line (TN1-3, TPM: 0). These results suggest that the expression level of the gene may be strongly influenced by individual differences. We also noticed no differences in the gene expression of potato allergen proteins (Sola t 1-4: Allergome, http://www.allergome.org/index.php) between the NN and TN lines. Thus, our results show that no clear transcriptomic differences were detected between the NN and TN lines.

### 3.3 Proteomic Analysis of Tubers from NN- and TN-grafted Potato Plants

**Fig. 3.**
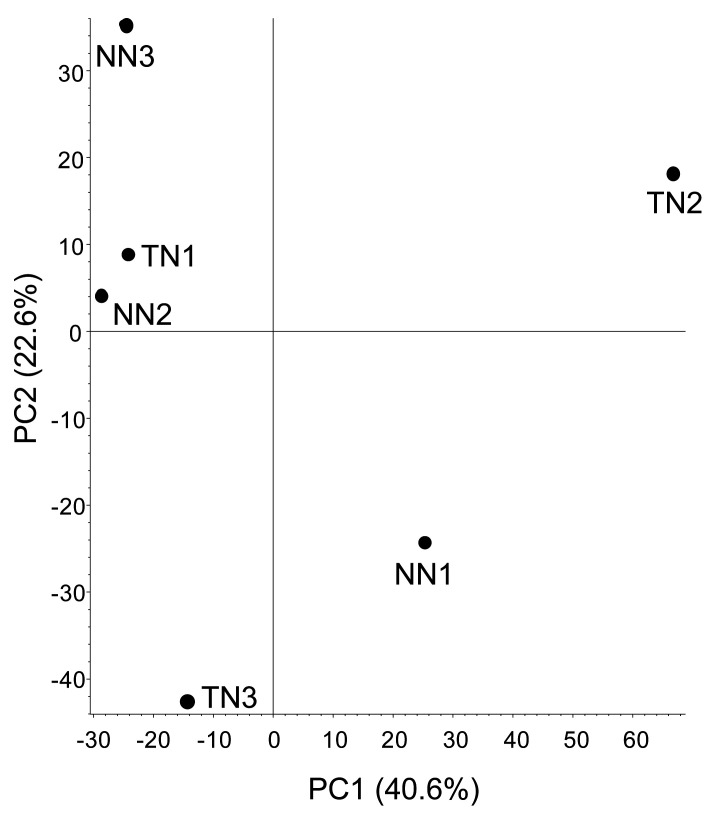
Principal component analysis of proteome data for the NN and TN lines. Each plot represents an individual sample of three biological replicates. Percentage values in parentheses indicate the respective contribution ratios.

Our proteomic dataset showed that 3,747 proteins were identified. PCA was performed on the accumulation data of these proteins for each sample (**Fig. 3**). These results revealed that no clusters formed for each line. This result suggests that there were no clear differences in protein accumulation between the NN and TN lines. Next, we attempted to identify proteins that showed significant differences in accumulation between the two lines. We identified 139 proteins from two or more fragments in each full-length amino acid sequence and had a *p*-value of less than 0.05. In addition, we also found 21 proteins whose abundance ratio was more than twice as high in the TN line than in the NN line (Supplementary Table S1). Among them, five proteins were identified as protease inhibitors (Uniprot Accession IDs: P58602, M1AMY6, M1AMY3, P24743, and M1AMY2). In contrast, we also identified ten proteins whose abundance ratio in the TN line was less than half that of the NN line (Supplementary Table S2). Of these, two proteins (M1C3M3 and M1BNZ2) were identified with esterase function. We also found that potato allergen proteins (Sola t 1-4) showed no difference in the abundance ratio between the NN and TN lines. Moreover, the StSP6A protein (M1C558) was not detected in either TN or NN tubers.

### 3.4 Metabolomic Analysis of Tubers from NN- and TN-grafted Potato Plants

Non-targeted metabolomic analyses were performed using LC-ESI-MS and GC-EI-MS to clarify whether FT-expressing potato scions may have influenced the metabolite compositions of tubers harvested from the rootstocks of non-transgenic potato plants.

**Fig. 4.**
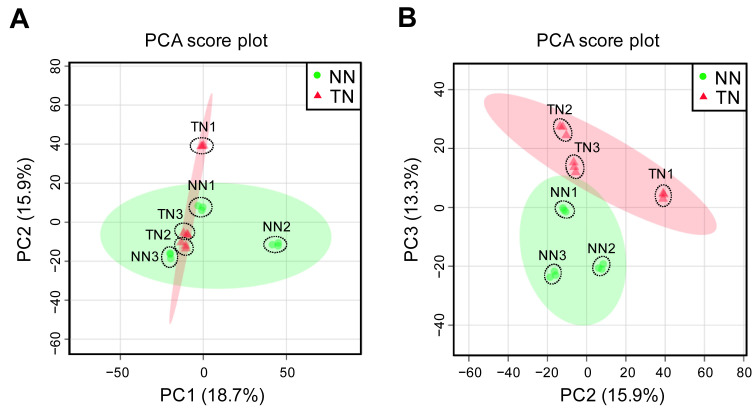
Accumulation profiles of hydrophilic metabolites in tubers from rootstocks grafted to FT-expressing potato scions. Metabolic profiling was performed using LC-ESI(+)-MS. PCA score plot graphs show: (A) PC1 and PC2, and (B) PC2 and PC3. Each plot represents an individual sample (three biological replicates and three analytical replicates). Percentage values in parentheses indicate the respective contribution ratios. The 95% confidence regions for the NN and TN lines are shown as light red and light green ovals, respectively.

LC-ESI(+)-MS analysis of the methanol:water (4:1, v/v) extracts of potato tuber samples was performed to study the accumulation profiles of hydrophilic compounds in the TN and NN lines. A series of data filtering processes identified 2,428 ion peaks as metabolite-candidates. To compare the metabolic profiles between the TN and NN lines, we performed a PCA on a data matrix consisting of 2,365 ion peaks versus samples with ion intensity as the variable value. A two-dimensional PCA score plot for PC1 and PC2 showed no decisive cluster separation between the TN and NN lines (Fig. 4A). When we focused on individual samples, we found that the scores of NN2 and TN1 were slightly higher than the other samples with respect to the PC1 axis and the PC2 axis, respectively. The ten highest positive and negative loadings for component 2 are shown in Supplementary Table S3. The compound annotations of the ion peaks listed in Supplementary Table S3 did not match with potato steroid glycoalkaloids, α-solanine (formula: C45H73NO15, exact mass: 867.498), or α-chaconine (formula: C45H73NO14, exact mass: 852.503). A two-dimensional PCA score plot for PC2 and PC3 showed loose cluster separation between the TN and NN lines (Fig. 4B), although the individual samples within each line were not tightly clustered. Among the 2,428 ion peaks, only 63 (2.6%) showed more than a two-fold difference in mean ion intensity (P_FDR_ < 0.05) between the TN and NN lines, whereas the other 2,365 ion peaks (97.4%) showed no significant difference in mean ion intensity between lines. Moreover, all 63 of the ion peaks that did show a difference in ion intensity were detected more than one sample in both lines. Of these 63 ion peaks, 13 and 50 showed significantly higher and lower mean ion intensities in the TN line than in the NN line, respectively (Supplementary Tables S4 and S5). The estimated chemical formulas for these ion peaks are shown in Supplementary Tables S4 and S5; however, there were no compound annotations corresponding to potato steroid glycoalkaloids, α-solanine, or α-chaconine.

**Fig. 5.**
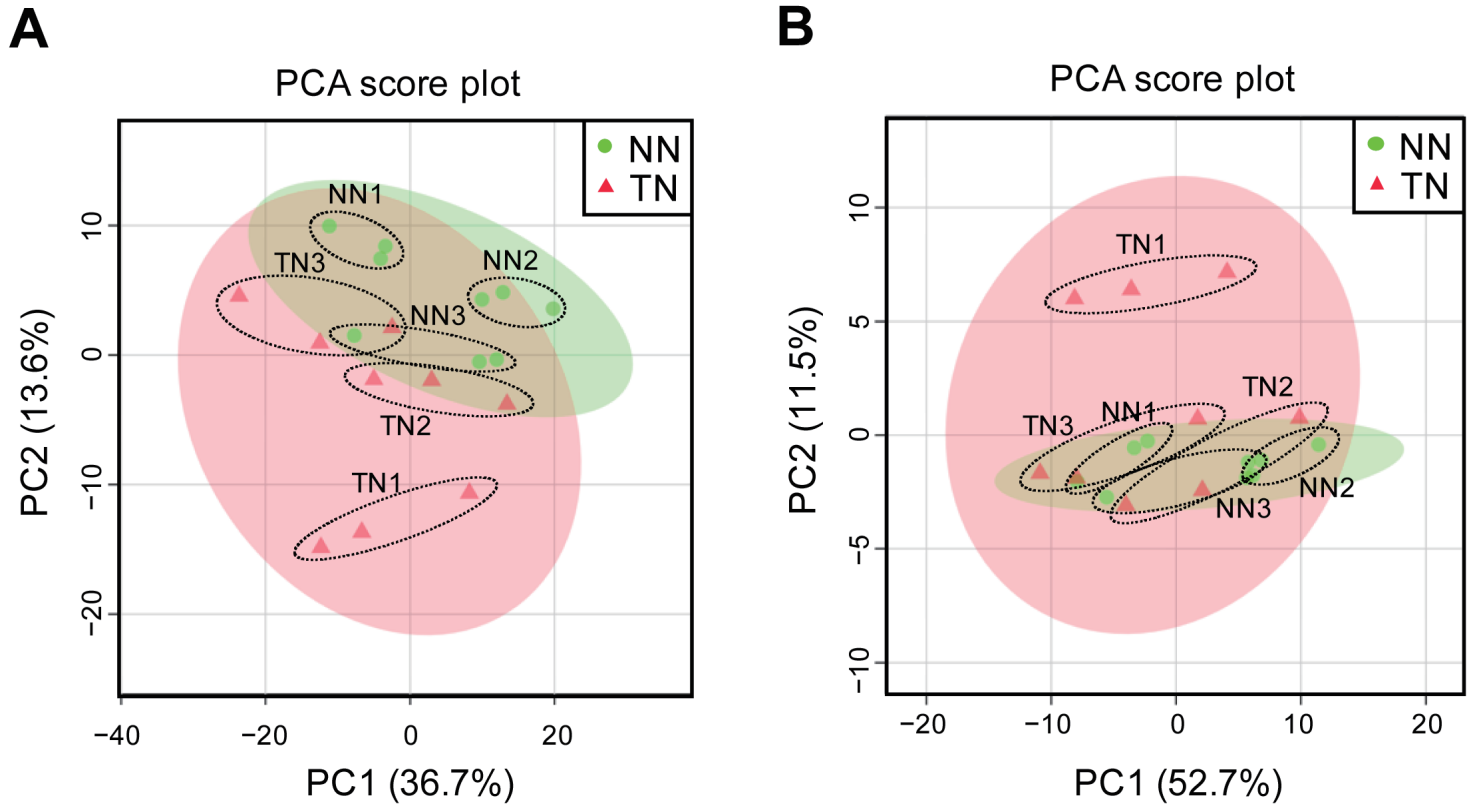
Lipid accumulation profiles of tubers from rootstocks grafted to FT-expressing potato scions. Metabolic profiling was performed using LC-ESI(+/−)-MS. Shown are PCA score plot graphs obtained using (A) positive ion mode and (B) negative ion mode. Each plot represents an individual sample (three biological replicates and three analytical replicates). Percentage values in parentheses indicate the respective contribution ratios. The 95% confidence regions for the NN and TN lines are shown as light red and light green ovals, respectively.

Next, we performed LC-ESI(+/−)-MS analysis of the methanol: methyl *tert*-butyl ether (3:10, v/v) extracts of potato tuber samples to identify any lipids that were differentially accumulated between the TN and NN lines. A total of 370 and 88 lipid-derived ion peaks were selected from the analytical data obtained by the positive and negative ion modes, respectively. To compare the lipid accumulation profiles among TN and NN samples, we again performed a PCA using data matrix consisting of ion peaks and samples, with ion intensity as the variable value. A two-dimensional PCA score plot for PC1 and PC2 again showed no cluster separation between the TN and NN lines; this was true both for the positive ion mode (Fig. 5A) and the negative ion mode (Fig. 5B). However, we did find that the PC2 axis differentiated TN1 from all other samples in both modes (Fig. 5A-B). The ten highest positive and negative loadings for component 2 in Fig. 5A and Fig. 5B are shown in Supplementary Tables S6 and S7, respectively. The lipid accumulation profile for the TN1 extract was characterized by lipid classes such as TGs (triacylglycerides), Cer (ceramide), and PMe (phosphatidylmethanol). We also found that the compound annotations of the ion peaks (Supplementary Tables S6 and S7) did not match the predicted peaks for potato steroid glycoalkaloids, α-solanine, or α-chaconine. Only six ion peaks (ID 337, 455, 591, 696, 961, and 1150) from the data obtained by positive ion mode (1.3%) showed more than a two-fold increase in mean ion intensity in TN plants relative to NN plants at P_FDR_ < 0.05. These were annotated as AcHexSiE (acyl hexosyl sitosterol ester) 23:0, PE (phosphatidylethanolamine) 18:0_13:0, DG (diacylglyceride) 18:1_18:3, Cer 18:1;O3/26:0, Hex2Cer (dihexosylceramide) 21:1;O3/16:1;O, and DG 10:0_21:1 (Supplementary Table S8). No ion peak from the negative ion mode showed a significant difference in mean ion intensity between the TN and NN lines.

**Fig. 6.**
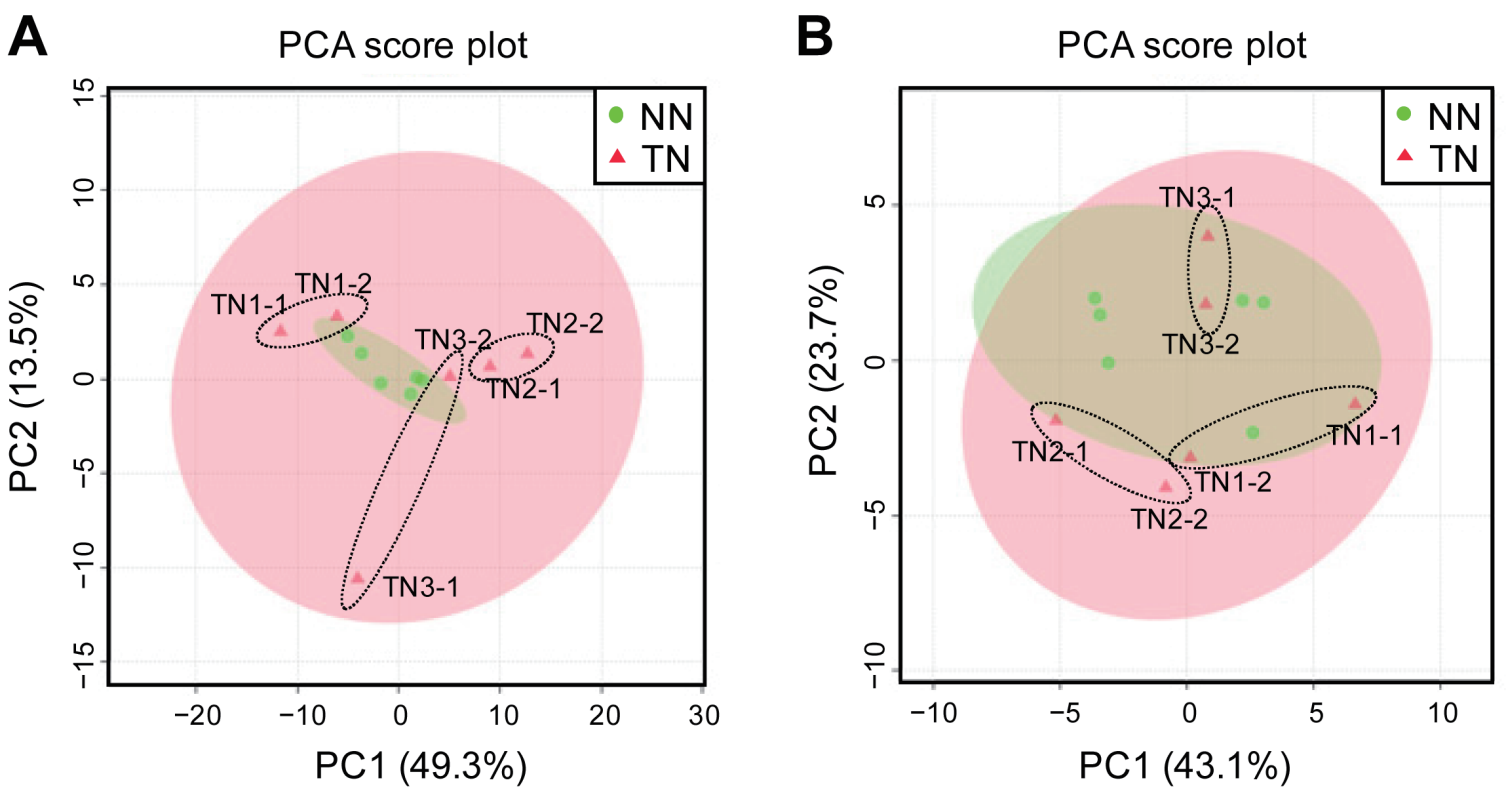
Comparison of the metabolite compositions of tubers from rootstocks grafted to FT-expressing potato scions. Metabolite accumulation profiles were obtained using GC-EI-MS. Shown are PCA score plot graphs obtained from polar fraction (A) and nonpolar fraction (B) data. Each plot represents an individual sample (three biological replicates and two analytical replicates). Percentage values in parentheses indicate the respective contribution ratios. The 95% confidence regions for the NN and TN lines are shown as light red and light green ovals, respectively.

Next, our GC-EI-MS analysis of potato tuber samples identified 112 and 38 metabolite peak groups from the polar and the nonpolar fractions, respectively, after peak deconvolution by AIoutput2. Each metabolite peak group represents a signal from a single metabolite. Using representative peak intensities from individual peak groups, we performed a PCA to once again compare metabolite composition between the NN and TN lines. The peak group identified as sucrose was excluded from the PCA because its peak intensity was saturated. In the polar fraction analysis, a 2D plot based on the principal component scores of PC1 (49.3% contribution) and PC2 (13.5% contribution) did not show an obvious cluster separation between the NN and TN lines (Fig. 6A), although TN3-1 did appear to be distinct from the NN samples, especially along the second principal component axis. The reason for this is unknown. In contrast, the TN3-2 metabolome was found directly within the NN cluster. None of the metabolites in the polar fraction showed more than a two-fold difference in relative abundance at P_FDR_ < 0.05 between the NN and TN lines (data not shown). For the analysis of the nonpolar fraction, a 2D plot based on the scores of PC1 (43.1% contribution) and PC2 (23.7% contribution) again failed to show cluster separation between samples from the NN and TN lines (Fig. 6B). Moreover, none of the metabolites in the nonpolar fraction differed more than 2-fold in relative abundance between the NN and TN lines at P_FDR_ < 0.05 (data not shown).

Potato tubers contain glycoalkaloids, which are toxic steroids that can be potentially harmful for human or animal consumption. In commercial potato cultivars, the main steroid glycoalkaloids in tubers are α-solanine and α-chaconine. To compare glycoalkaloid content in NN and TN tubers, we examined to the annotation of the ion peaks obtained by the LC-ESI(+)-MS analysis of the methanol:water (4:1, v/v) extracts. Here, we identified ion peaks No. 3848 and No. 3899 as possessing the molecular formulas corresponding to α-solanine and α-chaconine, respectively. As shown in Supplementary Fig. S1, we observed no significant differences in the peak area of ion peaks No. 3848 and No. 3899 between the NN and TN lines. These peak areas were higher in samples NN2 and TN1 than in the others (Supplementary Fig. S1). Taken together, our results indicate that the content of the SGAs, α-solanine and α-chaconine, were likely not affected by FT expression in the scions of the grafted plants, but instead varied naturally among individual plants and/or tubers.

### 3.5 Nutrient Composition Analysis

**Fig. 7.**
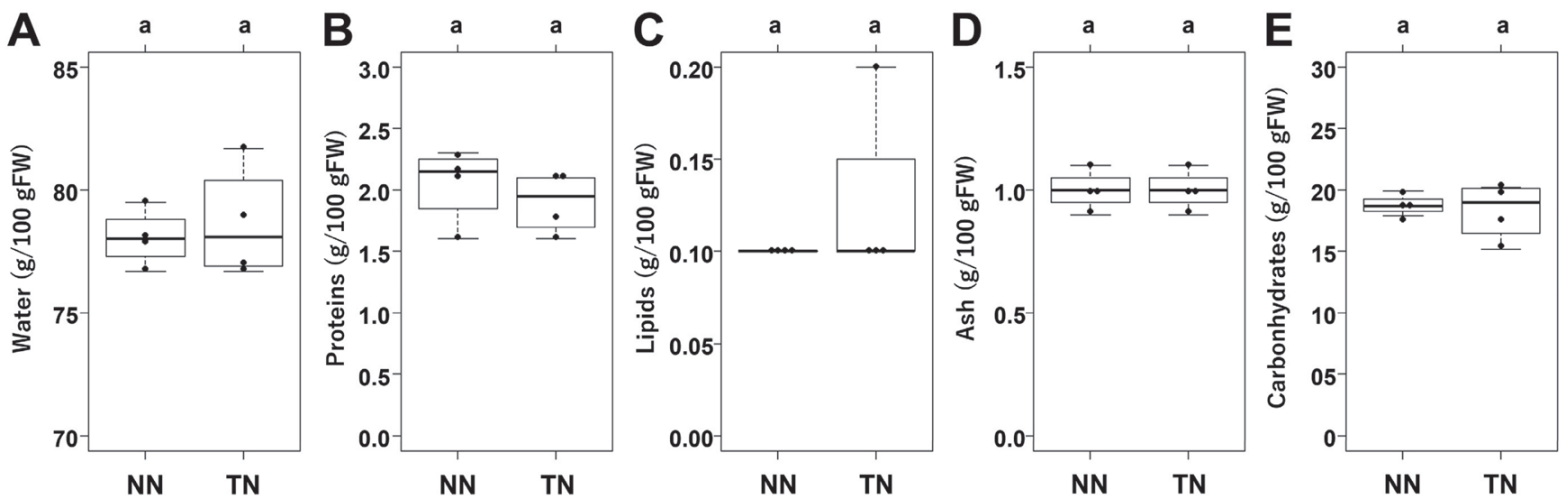
General food component analyses of tubers from non-transgenic potato rootstocks. Panels A-E display the water, protein, lipid, ash, and carbohydrate contents, respectively, of tubers from non-transgenic potato rootstocks scioned with non-transgenic scions (NN) and transgenic potato scions (TN). Different letters above the bars indicate significant differences among grafted plants as determined by Tukey HSD test (ɑ=0.05). Error bars indicate standard error.

Analyses of nutrients—i.e., water, protein, lipids, ash, and carbohydrates—were conducted by Japan Food Research Laboratories. Measured nutrient content from different samples is shown in **Figure 7**. No significant differences were observed for any nutrients when comparing the tubers of the TN- and NN-grafted plants (**Fig. 7**). These measurements closely approximated those found in the Standard Tables of Food Composition in Japan (Seventh Revised Version)^31^^)^. This result suggested that the nutrient composition of tubers from non-transgenic rootstocks were not affected by scion type or grafting operation.

## 4. Discussion

The existence of florigen, a plant hormone thought to regulate flowering by perceiving photoperiod condition, was first suggested in the 1930s. The nature of florigen remained unknown for the next 70 years, but in 2007 it was revealed that florigen consisted of proteins encoded by the *FLOWERING LOCUS T* (*FT*) gene in *Arabidopsis* and its rice homologue *Heading date 3a* (*Hd3a*)^32^^,^^33^^)^. FT is a globular protein of approximately 20 kDa and is now recognized as a multifunctional plant hormone. One of its most important applications is ectopic expression in fruit trees; transient expression of the *FT* gene permits the flowering of seedlings of fruit trees, which accelerates the fruit tree breeding process^34^^)^. However, FT is also involved in leaf morphogenesis and shoot growth^35^^,^^36^^)^, and is an important factor in cereal crop dormancy. The pre-harvest sprouting of cereals can cause economically significant losses, and the control of dormancy may prevent this from occurring. Therefore, the fine regulation of FT to control dormancy may be an attractive tool for the development of pre-harvest sprouting tolerance^37^^)^.

Because florigen is synthesized in response to photoperiod cues and is translocated from the leaves to the shoot apical meristem, the systemic transport mechanisms involved in FT signaling have been investigated using grafting techniques in various plants. To examine whether the flowering signal was translocated as an mRNA or as a protein, the distribution of *FT* mRNA and a fluorescent protein-fused FT protein was examined in *Arabidopsis*. *FT* mRNA was strongly detected in phloem companion cells, but not in the shoot apical meristem or protophloem. The FT protein was detected in both phloem tissue and the shoot apical meristem^32^^,^^38^^)^. Similar translocation of the FT protein was also observed in rape, tomato, squash, and rice^33^^,^^39^^,^^40^^,^^41^^)^. Therefore, the FT-linked flowering signal is translocated as the FT protein itself rather than the corresponding mRNA, and the FT protein moves through the phloem. In transgrafted plants, it has been reported that the FT protein moves from the rootstock to scion and vice versa. In *Arabidopsis*, when a transgenic scion expressing the *FT* gene was grafted onto an *FT*-deficient mutant rootstock, the FT protein produced in the scion was detected in the vasculature of the rootstock^32^^)^. It is thought that this mobility helps FT to promote tuber and bulb formation in potato and onion^42^^,^^43^^)^.

In previous reports, we investigated the effects of a GM rootstock on the mRNA, protein, and metabolite profiles of scions^11^^,^^12^^)^. Here, we investigated the effects of a GM scion on the transcriptomic, proteomic, and metabolomic profiles of non-GM rootstock. Because patterns of gene expression during tuberization can be epigenetically regulated by grafting with RdDM-inducing scions onto a non-GM potato rootstock^15^^)^, transgrafting in potato plants has the potential to be a key application of NPBT. However, to clarify whether or not transgrafting of potato presents risks, in this study we used various omics analyses to examine potato tubers harvested from transgrafted plants. As mentioned above, the FT protein can be translocated from scion to rootstock. Moreover, when GM scions transformed with the *StSP6A* gene were grafted onto a non-GM potato rootstock, tuber formation was reported to be promoted^44^^)^. However, in this study we observed a significant difference in total tuber yield per plant between non-GM rootstock grafted with TN and NN scions (Fig. 1D). Given that there was no significant difference in weight per tuber or in the number of tubers per plant, the observed difference in tuber weight per plant may be due to high inter-individual variability in TN. The stimulatory effects on tuberization observed in potato plants grafted with the *StSP6A*-transformed scion^44^^)^ was not clearly observed in our results. The reason for this inconsistency may be explained by the timing of the culturing and harvesting of transgrafted potato plants. Here, the transgrafted potato plants were cultivated for approximately three months from late January, and the tubers were harvested from late April to early May. This timing of cultivation was optimal for tuberization (short day condition), and the effects of FT produced by the scion were not clearly observed. In fact, the stimulatory effects of *StSP6A* overexpression on tuberization were observed under non-inductive, long day conditions^44^^)^. We also observed the transgrafted potato plants showed an increased accumulation of *StSP6A* mRNA in the transgenic body via the transgene expression (Supplementary Fig. S2) and enhanced tuberization in the rootstocks of the TN potato plants when the grafted plants were cultivated under long day conditions (16 h light, 8 h dark, Supplementary Fig. S3).

In this study, omics analyses (i.e., analyses of the transcriptome, proteome, and metabolome) were performed on tubers to evaluate whether there was any risk associated with the transgrafting of potato plants. Taken together our results showed no significant differences in the mRNA profiles of tubers from the TN and NN lines. Among 35,823 expressed genes, we identified only one differentially expressed gene. In contrast, we identified 21 and 10 proteins whose expression was increased and decreased in tubers by grafting with a GM scion, respectively. Among these differentially accumulated proteins, five were increased in TN tubers and were annotated as protease inhibitors. Although protease inhibitors are abundant in potato tubers, where they function as storage and defense proteins, these proteins have also been considered to have antinutrient effects on human consumption^45^^)^. The most abundant potato protease inhibitor is a serine protease inhibitor that is a member of the Knuitz family. In addition, the second most abundant protease inhibitor was a member of a family of potato cysteine protease inhibitors. These two classes of protease inhibitors represent 22% and 12% of the total proteins present in potato juice, respectively^46^^)^. Moreover, the five protease inhibitors whose expression increased in the TN tubers (Supplementary Table S1) were also among these highly abundant protease inhibitors. However, the total protein content of TN tubers was nearly the same as in NN tubers (Fig. 7). This result suggests that only relatively minor members of the serine or cysteine protease inhibitor families were increased in TN tubers. At present, we cannot provide a possible reason for the differential accumulation of these proteins between the TN and NN potato tubers.

Potato allergy is generally rare, and minor cases of potato allergy are known to be induced by patatin (Sola t 1), a highly abundant potato protein of 42 kDa^47^^)^. This protein was not included in the list of differentially accumulated proteins (Supplementary Tables S1 and S2).

Metabolic profiles also showed no apparent difference between tubers from the TN and NN lines. At most, FT expression in scions might affect the accumulation of some very minor metabolites of unknown identities in non-GM tubers (Supplementary Tables S4, S5, and S8). These metabolites remain unidentified despite the use of a cutting-edge metabolomics pipeline based on high-resolution MS analysis. Moreover, we also found that FT expression in scions did not influence the accumulation of the potato steroid glycoalkaloids α-solanine and α-chaconine. Finally, nutrient composition analysis showed that transgrafting also did not affect the nutritional value of potato tubers.

## 5. Conclusion

Plants synthesize a variety of secondary metabolites that act as forms of chemical defense against biotic and abiotic stresses under variable environmental conditions. Some of these plant-derived substances reduce food safety, but humans have successfully reduced the accumulation levels of such toxic substances during the breeding and cultivation of the ancestors of modern crop plants. However, when newly developed crops are produced with the aid of novel breeding techniques such as NPBT, it is imperative to ensure that the biosynthesis of harmful secondary metabolites remains low. From this point of view, it is essential to use multi-omics approaches to conduct metabolic profiling for food safety assessment and to individually measure the abundance of toxic secondary metabolites such as steroidal alkaloids (e.g., nicotine, α-tomatine, and α-solanine produced by Solanaceae plants).

The results of this study indicate that *FT* expression in GM potato scions did not adversely affect the accumulation levels of toxic metabolites and allergens in non-GM rootstock potato tubers. On the other hand, mobile small RNA molecules are known to exclusively move from shoots to roots^48^^,^^49^^)^, and the expected application of transgrafting in potato is grafting of RdDM-inducing GM scions onto non-GM rootstocks^15^^)^. Therefore, in such case, further evaluation of the food safety of small RNA-mediated changes in potato tubers is required.

## Supplementary materials
