# Supplementary material for: Omics Profiles of Non-GM Tubers from Transgrafted Potato with a GM Scion
Source: Food Saf (Tokyo). 2023 Feb 11;11(1):1–20. doi: 10.14252/foodsafetyfscj.D-22-00010 (PMC10034357; doi:10.14252/foodsafetyfscj.D-22-00010)
Supplement: Supplementary file 1 [file foodsafetyfscj-11-1-s001.pdf]

Supplementary materials

Supplementary methods

Cultivation condition in cultured room

Cutting of the transgenic (35:StSP6A #7) and non-transgenic potatoes were cultured on agar media for 3-5 weeks and transferred to plastic pots (10.5 cm in diameter) with the mixed soil consisted of peat moss, vermiculite, parlite, Genki-kun No.1 (Katakura Co-op Agri, Tokyo, Japan) at a ratio of 1:1:1:9, respectively. After the acclimatization process for a week, the plantlets were cultivated in a cultivation room with a temperature of 25°C, a 16-hour light period, and an 8-hour dark period. After 2-3 week cultivation, the grafting were performed. On two weeks after grafting, soils were added to a height of 2 cm above the graft points, and continued cultivation the same condition until 3 months after grafting.

Quantitative reverse-transcription PCR (qRT-PCR) for evaluation of transgene expression in transgrafted potato

The leaves and stolons of grafting potatoes were sampled and the total RNA extractions were performed with the RNeasy plant Mini Kit (Qiagen, Hilden, Germany) following the manufacturer's instruction. After RNA extraction, 1 μg of total RNA was subjected as a template for the reverse transcription reaction by using the PrimeScript RT reagent Kit with gDNA Eraser (Takara-Bio, Shiga, Japan). qRT-PCR was performed by using reagent of KOD SYBR® qPCR Mix (Toyobo, Osaka, Japan) and the instrument of Applied Biosystems 7900 HT Fast Real-Time PCR System (Thermo-Fisher Scientific) according to the following thermal cycle: pre-incubation at 98°C for 2 min followed by 40 cycles of denaturing at 98°C for 10 sec, annealing at 55°C for 10 sec and extension at 60°C for 30 sec, and a dissociation stage. The transcript level of a potato endogenous *Ubiquitin* gene was used as reference for quantitation. The primers used for qRT-PCR were as follows: 5'- TATATCATGGCCGACAAGCA -3' and 5'- GAACTCCAGCAGGACCATGT -3' for transgene of *StSP6A* and 5'- CTGGAAAGCAGCTCGAGGAT -3' and 5'- CCTGGATCTAGCCTGGACATTA -3' for *Ubiquitin* gene.

doi: 10.14252/foodsafetyfscj.D-22-00010

Supplementary Table S1. List of proteins with significantly increased protein abundance ratios in the TN line as identified by proteomic analysis.

Description	Uniprot Accession ID	Abundance ratio TN/NN
mRNA cap-binding protein	M1C6I4	100
Serine/threonine-protein kinase TOR	M1CF02	100
Uncharacterized protein	M1DLW4	7.48
UDP-glucose 4-epimerase	M1AWM7	4.92
Ribosomal protein S14	Q38JI8	4.63
Ribulose bisphosphate carboxylase large chain	P25079	3.65
* Cysteine protease inhibitor 4 (Fragment)	P58602	3.37
Uncharacterized protein	M1AM96	3.15
* Miraculin-like	M1AMY6	2.76
Trafficking protein particle complex subunit 6b	M1CMT7	2.72
NADH-ubiquinone oxidoreductase 19 kDa subunit	M1C2U2	2.63
* Cysteine protease inhibitor 1	M1AMY3	2.63
* Serine protease inhibitor 8 (Fragment)	P24743	2.48
Prolactin regulatory element-binding protein	M1A6I5	2.42
Phospholipase A1	M1AQA0	2.35
* Cysteine protease inhibitor 9-like	M1AMY2	2.25
Subtilase	M1AP96	2.21
Catechol oxidase	M1BMR5	2.17
SAP domain containing protein	M1CMR2	2.15
Catechol oxidase	M1CW46	2.14
Divinyl ether synthase	M1CCW0	2.01

* Proteins with an asterisk are classified as protease inhibitors.

Supplementary Table S2. List of proteins with significantly increased protein abundance ratios in the NN line as a result of proteome analysis.

Description	Uniport Accession ID	Abundance ratio TN/NN
Nucellin	M1D345	0.01
Nucleolar phosphoprotein	M1AHF2	0.16
Apyrase	P80595	0.31
* Polyneuridine-aldehyde esterase	M1C3M3	0.39
Superoxide dismutase	M1CMZ3	0.39
Embryo-specific 3	M1C755	0.43
30S ribosomal protein S1	M1A029	0.43
Cysteine protease inhibitor 9	M1AMY9	0.48
60S acidic ribosomal protein	M1AUE9	0.49
* Pectinesterase	M1BNZ2	0.5

* Proteins with an asterisk are classified as esterase.

Supplementary Table S3. The ten highest positive and negative loadings of component 2 from a PCA based on the LC-MS analysis of hydrophilic extracts (corresponds to the score plot graph in **Fig. 4**).

Ion peak ID	RT[a]	*m/z*[b]	Adduct[c]	Exact mass[d]	Database[e]	Formula[f]	Loadings (PC2)	Mean ion intensity ratio (TN1/NN)	Mean ion intensity ratio (TN2/NN)	Mean ion intensity ratio (TN3/NN)
4560	19.18	183.989	$[M+H]^+$	182.981	EX-HR2	C7H5O1N1S2	**0.04860**	1.9	1.1	1.1
3472	15.27	545.384	$[M+H]^+$	544.377	UC2	C33H52O6	**0.04830**	2.0	0.8	0.9
3473	15.27	401.342	$[M+H]^+$	400.334	UC2	C27H44O2	**0.04765**	1.9	0.8	0.8
3471	15.27	743.458	$[M+H]^+$	742.451	UC2	C39H66O13	**0.04744**	1.8	0.6	0.9
3474	15.27	383.331	$[M+H]^+$	382.324	UC2	C27H42O	**0.04689**	1.9	0.9	1.0
707	8.51	446.152	$[M+H]^+$	445.145	EX-HR2	C17H35O4N1S4	**0.04614**	2.0	0.9	1.0
327	4.07	171.113	$[M+H]^+$	170.106	UC2	C8H14O2N2	**0.04577**	2.2	0.9	1.4
1166	10.11	278.607	$[M+2H]^{2+}$	555.200	UC2	C16H41O6N7S4	**0.04455**	3.0	0.0	1.8
2204	12.43	393.152	$[M+H]^+$	392.145	EX-HR2	C24H24O3S1	**0.04454**	1.6	0.9	0.9
266	3.85	442.054	$[M+H]^+$	441.047	UC2	C16H15O10N3S	**0.04453**	2.5	0.0	0.6
2267	12.58	421.147	$[M+H]^+$	420.140	EX-HR2	C9H26O7N8P2	**-0.04469**	0.3	1.2	1.1
405	4.51	221.092	$[M+H]^+$	220.085	UC2	C11H12O3N2	**-0.04569**	0.5	1.2	1.0
2442	12.92	335.134	$[M+H]^+$	334.126	UC2	C15H18O5N4	**-0.04615**	0.4	1.0	1.1
954	9.52	125.071	$[M+H]^+$	124.064	UC2	C6H8ON2	**-0.04661**	0.3	1.2	1.2
2446	12.93	177.112	$[M+H]^+$	176.105	EX-HR2	C8H16O4	**-0.04692**	0.3	1.1	1.3
2103	12.19	335.134	$[M+H]^+$	334.126	UC2	C15H18O5N4	**-0.04717**	0.3	1.1	1.3
2447	12.93	159.102	$[M+H]^+$	158.094	UC2	C8H14O3	**-0.04752**	0.4	1.0	1.2
2106	12.19	159.102	$[M+H]^+$	158.094	UC2	C8H14O3	**-0.04765**	0.2	1.1	1.3
2449	12.93	353.144	$[M+H]^+$	352.137	EX-HR2	C14H24O10	**-0.04802**	0.1	1.0	1.2
2445	12.93	370.171	$[M+H]^+$	369.164	EX-HR2	C9H24O5N9P1	**-0.04886**	0.3	1.1	1.2

[a]Mean retention time (min) of the analyte.
[b]Measured *m/z* of the analyte.
[c]Adduct ion of the analyte estimated by considering the mass differences between the ion of interest and all other ions in the corresponding mass spectrum.
[d]Calculated exact mass of the analyte by considering its measured *m/z* and adduct ion.
[e]The database that contained the record which showed minimal error to the calculated exact mass of the analyte. Database search was performed by MFSearcher system using the calculated exact mass of the analyte as a query. The databases used were ExactMassDB-HR2 (EX-HR2) and the Unique Connectivity of UnCharged compounds database (UC2). In-house standard compound database search was also performed considering the average *m/z*, RT, and MS/MS spectrum of each ion features, but no hits were found.
[f]The database that contained the record which showed minimal error for the calculated exact mass of the analyte.

Supplementary Table S4. List of ion peaks with significantly higher mean ion intensities in TN than in NN.

Ion peak ID	RT[a]	*m/z*[b]	Adduct[c]	Exact mass[d]	Database[e]	Formula[f]	Mean ion intensity (NN)	SD (NN)	Mean ion intensity (TN)	SD (TN)	Mean ion intensity ratio (TN/NN)	p-value	q-value
375	4.41	223.021	$[M+H]^+$	222.013	EX-HR2	C6H10O3N2S2	4374	4131	18494	5623	4.2	1.6E-05	2.4.E-03
710	8.52	129.055	$[M+H]^+$	128.047	UC2	C6H8O3	15852	12730	40270	11780	2.5	6.5E-04	1.6.E-02
1268	10.34	418.228	$[M+H]^+$	417.221	EX-HR2	C21H33N5P2	38397	16678	77596	31815	2.0	4.8E-03	3.9.E-02
1272	10.37	423.184	$[M+H]^+$	422.177	EX-HR2	C17H27O1N8P1S1	9441	4258	20018	6388	2.1	7.8E-04	1.7.E-02
1439	10.72	277.105	$[M+H]^+$	276.098	EX-HR2	C8H21O8P1	10747	5307	23782	7464	2.2	5.9E-04	1.4.E-02
3406	15.12	515.119	$[M+H]^+$	514.112	UC2	C25H22O12	6503	9169	17100	2791	2.6	4.4E-03	4.9.E-02
3715	15.82	1090.717	$[M+4H]^{4+}$	4358.838	no hit	unknown	18654	10674	44180	17966	2.4	2.1E-03	3.2.E-02
3865	16.17	1064.959	$[M+4H]^{4+}$	4255.806	no hit	unknown	55154	42434	164472	56104	3.0	2.6E-04	1.1.E-02
3871	16.17	852.374	$[M+H]^+$	851.366	EX-HR2	C36H71O11N1P2S3	201893	299886	1210758	346316	6.0	6.0E-06	1.9.E-03
4195	16.93	1126.796	$[M+H]^+$	1125.789	EX-HR2	C53H132O2N3P5S4	6144	8550	29736	18792	4.8	3.4E-03	4.2.E-02
4210	16.96	1014.367	$[M+H]^+$	1013.360	EX-HR2	C52H68N7P1S6	5044	8060	33229	19315	6.6	9.5E-04	2.3.E-02
4431	18.09	486.291	$[M+2H]^{2+}$	970.568	EX-HR2	C55H88O8P2S1	5978	3226	17270	6697	2.9	3.2E-04	1.2.E-02
4746	20.55	274.274	$[M+H]^+$	273.267	EX-HR2	C16H35O2N1	3907	1657	28387	11293	7.3	8.3E-06	1.9.E-03

[a]Mean retention time (min) of the analyte.
[b]Measured *m/z* of the analyte.
[c]Adduct ion of the analyte estimated by considering the mass differences between the ion of interest and all other ions in the corresponding mass spectrum.
[d]Calculated exact mass of the analyte by considering its measured *m/z* and adduct ion.
[e]The database that contained the record which showed minimal error regarding the calculated exact mass of the analyte. Database searches were performed by the MFSearcher system using the calculated exact mass of the analyte as a query. The databases used were ExactMassDB-HR2 (EX-HR2) and the Unique Connectivity of UnCharged compounds database (UC2). In-house standard compound database search was also performed considering the average *m/z*, RT, and MS/MS spectrum of each ion features, but no hit was found.
[f]Chemical formula of the database record that showed minimal error for the calculated exact mass of the analyte.

Supplementary Table S5. List of ion peaks with significantly lower mean ion intensities in the TN line than in the NN line.

Ion peak ID	RT[a]	*m/z*[b]	Adduct[c]	Exact mass[d]	Database[e]	Formula[f]	Mean ion intensity (NN)	SD (NN)	Mean ion intensity (TN)	SD (TN)	Mean ion intensity ratio (TN/NN)	p-value	q-value
356	4.30	121.040	$[M-H_2O+H]^+$	138.043	UC2	C6H6O2N2	44032	36775	1483	2317	0.03	3.2.E-03	4.0.E-02
415	4.61	132.063	$[M+H]^+$	131.055	no hit	Unknown	117140	66278	24742	48051	0.21	3.8.E-03	4.4.E-02
418	4.63	133.099	$[M+H]^+$	132.092	no hit	Unknown	352957	72472	116584	178407	0.33	2.0.E-03	3.1.E-02
545	5.85	143.034	$[M-H_2O+H]^+$	160.037	UC2	C6H8O5	21824	6823	8044	5942	0.37	3.2.E-04	1.2.E-02
839	9.10	487.192	$[M+H]^+$	486.185	EX-HR2	C14H38O8N4S3	152350	65647	67544	35923	0.44	3.7.E-03	4.3.E-02
937	9.45	310.129	$[M-H_2O+H]^+$	327.132	UC2	C15H21O7N	48928	10307	21858	5046	0.45	2.6.E-06	1.1.E-03
1164	10.10	357.118	$[M+H]^+$	356.111	EX-HR2	C16H20O9	46780	17102	20344	4424	0.43	3.7.E-04	1.3.E-02
1165	10.10	170.081	$[M+H]^+$	169.074	UC2	C8H11O3N	81832	38476	36402	9835	0.44	3.4.E-03	4.2.E-02
1319	10.50	97.029	$[M+H]^+$	96.022	UC2	C5H4O2	14780	4099	7018	3300	0.47	4.2.E-04	1.3.E-02
1323	10.50	137.023	$[M+H]^+$	136.016	EX-HR2	C7H4O3	20440	4217	8780	5991	0.43	2.1.E-04	8.3.E-03
1878	11.66	263.139	$[M+H]^+$	262.132	UC2	C14H18O3N2	32475	8923	11178	7041	0.34	3.8.E-05	3.3.E-03
2016	11.97	166.050	$[M+H]^+$	165.043	UC2	C8H7O3N	68356	28026	28067	11782	0.41	1.1.E-03	2.0.E-02
2021	12.00	148.039	$[M+H]^+$	147.032	UC2	C8H5O2N	26108	8048	9069	6224	0.35	1.2.E-04	7.5.E-03
2150	12.31	177.055	$[M+H]^+$	176.047	UC2	C10H8O3	262715	33446	68885	103459	0.26	6.5.E-05	3.8.E-03
2379	12.77	658.214	$[M+H]^+$	657.206	EX-HR2	C35H36O6N3P1S1	22606	10577	7390	6833	0.33	2.3.E-03	3.4.E-02
2438	12.91	321.192	$[M+H]^+$	320.185	UC2	C16H24O3N4	42598	15078	19903	7361	0.47	9.1.E-04	1.8.E-02
2988	14.20	120.045	$[M+H]^+$	119.037	UC2	C7H5ON	17054	3479	6487	1588	0.38	3.5.E-07	3.1.E-04
3470	15.27	193.050	$[M+H]^+$	192.042	UC2	C10H8O4	32821	17216	7721	7396	0.24	9.9.E-04	2.0.E-02
4470	18.42	250.144	$[M+H]^+$	249.137	UC2	C14H19O3N	21449	9137	9529	2438	0.44	1.6.E-03	2.7.E-02
4474	18.45	623.239	$[M-H_2O+H]^+$	640.243	EX-HR2	C23H47O3N8P3S2	53912	20659	24076	15731	0.45	3.3.E-03	4.1.E-02
4635	19.38	213.149	$[M+H]^+$	212.141	UC2	C12H20O3	41256	8954	19226	9118	0.47	9.3.E-05	6.8.E-03
4669	19.61	294.214	$[M+H]^+$	293.207	no hit	Unknown	36985	3763	12631	11389	0.34	1.6.E-05	2.0.E-03
4690	19.85	269.201	$[M+H]^+$	268.194	EX-HR2	C18H24N2	45735	7118	4067	6135	0.09	4.6.E-10	6.2.E-07
4833	20.88	257.190	$[M+H]^+$	256.183	UC2	C18H24O	20695	7165	10067	6453	0.49	4.5.E-03	4.9.E-02
4901	21.46	309.206	$[M+H]^+$	308.199	UC2	C18H28O4	102046	29781	50346	13622	0.49	2.2.E-04	1.0.E-02
5001	22.54	157.122	$[M+H]^+$	156.115	UC2	C9H16O2	19076	9961	3721	4649	0.20	6.9.E-04	1.8.E-02
5011	22.66	294.214	$[M+H]^+$	293.207	no hit	Unknown	20056	7996	9797	2631	0.49	2.1.E-03	3.2.E-02
5063	23.08	137.096	$[M+H]^+$	136.089	UC2	C9H12O	19695	8787	2580	5579	0.13	1.5.E-04	8.0.E-03
5101	23.34	257.190	$[M-(Pentose-H_2O)+H]^+$	388.225	UC2	C23H32O5	19400	10481	4925	6675	0.25	3.0.E-03	3.8.E-02
5142	23.78	597.304	$[M+H]^+$	596.297	UC2	C34H44O9	181118	106455	57137	29029	0.32	3.9.E-03	4.5.E-02
5148	23.82	149.133	$[M+H]^+$	148.125	UC2	C11H16	23280	8265	11373	2567	0.49	7.9.E-04	1.7.E-02
5221	24.24	1039.673	$[M+H]^+$	1038.666	EX-HR2	C41H117N8P3S7	59731	46899	3908	5045	0.07	2.7.E-03	3.6.E-02
5240	24.40	478.293	$[M+H]^+$	477.286	UC2	C23H44O7NP	231409	133908	54092	18006	0.23	1.2.E-03	2.2.E-02
5260	24.59	109.102	$[M+H]^+$	108.094	UC2	C8H12	56259	16934	24494	23583	0.44	4.7.E-03	5.0.E-02
5263	24.59	520.340	$[M+H]^+$	519.333	UC2	C26H50O7NP	346653	205514	97633	34000	0.28	2.5.E-03	3.5.E-02
5272	24.78	360.274	$[M+H]^+$	359.267	UC2	C19H37O5N	53645	33634	11682	14446	0.22	3.4.E-03	4.2.E-02
5275	24.78	346.259	$[M+H]^+$	345.252	UC2	C18H35O5N	260402	132637	72708	75729	0.28	2.0.E-03	3.1.E-02
5278	24.78	211.133	$[M+H]^+$	210.126	UC2	C12H18O3	192598	100835	58075	54154	0.30	2.8.E-03	3.7.E-02
5280	24.79	370.259	$[M+H]^+$	369.252	EX-HR2	C20H35O5N1	51521	30465	12595	15556	0.24	3.6.E-03	4.3.E-02
5283	24.84	193.122	$[M+H]^+$	192.115	UC2	C12H16O2	22878	14756	4388	7276	0.19	3.9.E-03	4.8.E-02
5309	25.11	227.064	$[M+H]^+$	226.056	EX-HR2	C13H10N2S1	93476	18104	15301	22011	0.16	3.8.E-07	3.1.E-04
5331	25.21	346.259	$[M+H]^+$	345.252	UC2	C18H35O5N	33978	21780	7200	10963	0.21	4.6.E-03	5.0.E-02
5429	26.23	263.237	$[M+H]^+$	262.230	UC2	C18H30O	19792	9970	4895	3422	0.25	6.2.E-04	1.6.E-02
5433	26.24	534.364	$[M+H]^+$	533.357	EX-HR2	C22H66N1P3S3	52377	21881	18143	10887	0.35	6.8.E-04	1.6.E-02
5435	26.24	355.284	$[M+H]^+$	354.277	UC2	C21H38O4	39006	18011	13613	7282	0.35	1.2.E-03	2.2.E-02
5465	26.43	338.269	$[M+H]^+$	337.262	UC2	C20H35O3N	19559	8162	8704	5390	0.44	4.2.E-03	4.8.E-02
5634	27.72	645.366	$[M+H]^+$	644.359	UC2	C36H52O10	21449	5561	7637	6524	0.36	1.8.E-04	8.8.E-03
5659	27.94	295.227	$[M+H]^+$	294.220	UC2	C18H30O3	1190142	232866	458028	275905	0.38	1.6.E-05	1.9.E-03
5707	28.75	297.242	$[M+H]^+$	296.235	UC2	C18H32O3	43988	14124	19021	10562	0.43	6.2.E-04	1.6.E-02
5756	29.41	366.300	$[M+H]^+$	365.293	UC2	C22H39O3N	47240	18936	11487	9073	0.24	1.1.E-04	7.5.E-03

[a]Mean retention time (min) of the analyte.
[b]Measured *m/z* of the analyte.
[c]Adduct ion of the analyte estimated by considering the mass differences between the ion of interest and all other ions in the corresponding mass spectrum.
[d]Calculated exact mass of the analyte by considering its measured *m/z* and adduct ion.
[e]The database that contained the record which showed minimal error regarding the calculated exact mass of the analyte. Database searches were performed by the MFSearcher system using the calculated exact mass of the analyte as a query. The databases used were ExactMassDB-HR2 (EX-HR2) and the Unique Connectivity of UnCharged compounds database (UC2). In-house standard compound database search was also performed considering the average *m/z*, RT, and MS/MS spectrum of each ion feature, but no hit was found.
[f]Chemical formula of the database record that showed minimal error for the calculated exact mass of the analyte.

Supplementary Table S6. The ten highest positive and negative loadings of component 2 of the PCA based on the LC-ESI(+)-MS analysis of lipid extracts (corresponds to the score plot graph in **Fig. 5A**).

Ion peak ID	RT[a]	*m/z*[b]	Lipid class[c]	Acyl chain[d]	Adduct[e]	m-Score[f]	Grade[g]	Loadings (PC2)	Mean ion intensity ratio (TN1/NN)	Mean ion intensity ratio (TN2/NN)	Mean ion intensity ratio (TN3/NN)
1131	46.72	912.802	TG	19:1_18:2_18:2	$[M+NH_4]^+$	63.81	A	**0.1268**	0.7	0.9	1.0
1212	47.91	904.832	TG	18:0_18:1_18:1	$[M+NH_4]^+$	31.17	A	**0.1235**	0.4	0.6	1.0
1187	47.48	874.787	TG	16:0_18:1_18:2	$[M+NH_4]^+$	84.22	A	**0.1206**	0.5	0.6	1.1
1198	47.63	926.818	TG	20:0_18:2_18:3	$[M+NH_4]^+$	80.32	A	**0.1202**	0.5	0.7	0.9
1214	47.91	876.802	TG	18:0_16:0_18:2	$[M+NH_4]^+$	85.31	A	**0.1166**	0.5	0.6	1.1
1210	47.91	902.818	TG	18:0_18:1_18:2	$[M+NH_4]^+$	76.33	A	**0.1102**	0.5	0.5	1.0
1227	48.03	954.850	TG	22:0_18:2_18:3	$[M+NH_4]^+$	80.10	A	**0.1066**	0.4	0.6	0.9
1188	47.49	848.771	TG	16:0_16:0_18:2	$[M+NH_4]^+$	85.48	A	**0.1060**	0.7	0.6	1.2
1241	48.41	982.881	TG	24:0_18:2_18:3	$[M+NH_4]^+$	71.49	A	**0.1024**	0.2	0.0	1.0
1236	48.31	904.834	TG	20:0_16:0_18:2	$[M+NH_4]^+$	63.03	A	**0.1017**	0.4	0.3	1.0
1116	46.64	924.802	TG	20:2_18:2_18:2	$[M+NH_4]^+$	59.14	A	**-0.0809**	1.2	1.0	1.1
873	43.94	666.640	Cer	18:1;O3/24:0	$[M+H]^+$	27.84	B	**-0.0842**	1.3	1.1	1.1
753	42.08	572.541	CE	11:0	$[M+NH_4]^+$	7.77	C	**-0.0859**	1.5	1.0	1.3
927	44.47	710.666	Cer	18:1;O3/26:0;O	$[M+H]^+$	30.85	A	**-0.0883**	2.2	0.5	1.1
370	28.83	728.523	PE	17:1_18:2	$[M+H]^+$	23.48	C	**-0.0918**	3.0	1.2	1.7
285	26.33	726.508	PE	21:0_12:1	$[M+Na]^+$	22.85	B	**-0.1034**	4.6	2.1	2.3
942	44.70	844.703	AcHexCmE	18:1	$[M+NH_4]^+$	20.24	A	**-0.1044**	1.5	0.9	0.9
961	44.87	694.671	Cer	18:1;O3/26:0	$[M+H]^+$	22.11	B	**-0.1093**	14.8	11.9	4.5
930	44.51	646.578	Cer	18:1;O2/21:0;O	$[M+Na]^+$	8.81	D	**-0.1172**	2.7	1.6	0.8
872	43.94	868.703	AcHexChE	21:3	$[M+NH_4]^+$	13.50	A	**-0.1181**	2.4	0.8	0.8

[a]Mean retention time (min) of the analyte.

[b]Measured *m/z* of the analyte.

[c]TG, triacylglycerides; Cer, ceramides; CE, cholesterol esters; PE, phosphatidylethanolamines, AcHexCmE, acyl hexosyl campesterol esters; AcHexChE, acyl hexosyl sitosterol esters.

[d]In TG, CE, PE, AcHexCmE, and AcHexChE, acyl chains were annotated as "C-atoms:double bond equivalents", and acyl chains were separated by a separator "_" which means that the *sn*-position of acyl constituents is not known. In Cer, the sphingoid backborn was annotated as "C-atoms:double bond equivalents;O-atoms", and separated by a slash "/" from the *N*-linked fatty acid annotated as "C-atoms:double bond equivalents" or "C-atoms:double bond equivalents;O-atoms".

[e]Adduct ion of the analyte estimated by considering the mass differences between the ion of interest and all other ions in the corresponding mass spectrum.

[f]A match between the analyte and a Lipid Search database record was calculated by considering the number of product ions that matched. A higher m-Score indicates a more reliable annotation result.

[g]Degree of the structural estimation of the analyte. "Grade A" shows that both the lipid class and fatty acid chain(s) of the analyte are fully identified. "Grade B" shows that the tandem mass spectrum of the analyte contains both the lipid class-specific product ion and some fatty acid-derived product ions. "Grade C" shows that the tandem mass spectrum of the analyte contains either the lipid class-specific product ion or some fatty acid-derived product ions.

Supplementary Table S7. The ten highest positive and negative loadings of component 2 of a PCA based on the LC-ESI(−)-MS analysis of lipid extracts (corresponds to the score plot graph in **Fig. 5B**).

Ion peak ID	RT[a]	*m/z*[b]	Lipid class[c]	Acyl chain[d]	Adduct[e]	m-Score[f]	Grade[g]	Loadings (PC2)	Mean ion intensity ratio (TN1/NN)	Mean ion intensity ratio (TN2/NN)	Mean ion intensity ratio (TN3/NN)
151	28.85	709.484	PMe	18:2_18:2	$[M-H]^-$	33.18	B	**0.24501**	2.7	1.1	1.4
113	26.33	707.468	PMe	18:3_18:2	$[M-H]^-$	25.47	B	**0.23344**	7.2	2.5	3.2
247	34.73	598.507	Cer	18:1;O3/16:0	$[M+HCOO]^-$	22.76	C	**0.23144**	2.7	0.0	0.7
260	35.90	713.515	PMe	18:0_18:2	$[M-H]^-$	17.86	B	**0.2177**	2.3	0.6	1.1
196	31.61	685.483	PMe	16:0_18:2	$[M-H]^-$	28.14	B	**0.21345**	1.9	0.9	1.2
152	28.88	683.468	PMe	16:0_18:3	$[M-H]^-$	25.46	B	**0.20171**	2.4	1.0	1.6
325	43.45	726.628	Cer	17:1;O3/25:0;O	$[M+HCOO]^-$	20.98	B	**0.16505**	1.5	0.7	1.1
186	30.90	760.560	HexCer	18:1;O2/16:0;O	$[M+HCOO]^-$	14.27	C	**0.14893**	1.6	0.8	1.3
107	25.97	857.521	PI	18:2_18:2	$[M-H]^-$	66.12	A	**0.14546**	1.6	0.8	1.1
303	42.08	616.533	Cer	17:0;O/21:4	$[M+HCOO]^-$	8.00	D	**0.12297**	1.8	1.0	1.7
145	28.63	959.597	DGDG	16:0_18:3	$[M+HCOO]^-$	28.54	A	**-0.14193**	0.0	1.0	2.2
17	3.66	723.383	DGMG	18:2	$[M+HCOO]^-$	12.29	B	**-0.14211**	0.0	1.3	2.9
275	37.37	810.529	PS	20:2_18:2	$[M-H]^-$	6.61	C	**-0.14869**	0.9	0.9	1.1
337	43.69	1027.748	SQDG	31:0_18:2	$[M-H]^-$	6.74	D	**-0.15131**	0.8	1.1	1.4
177	30.00	712.494	PE	16:0_18:3	$[M-H]^-$	46.98	A	**-0.15383**	0.9	1.1	1.0
314	42.85	902.696	HexCer	18:1;O3/25:0;O	$[M+HCOO]^-$	11.06	C	**-0.15409**	0.8	0.8	1.0
34	4.87	561.330	MGMG	18:2	$[M+HCOO]^-$	10.86	B	**-0.16568**	0.2	0.9	1.2
150	28.73	800.547	PC	16:0_18:3	$[M+HCOO]^-$	54.27	A	**-0.20746**	0.8	1.1	1.1

doi: 10.14252/foodsafetyfscj.D-22-00010

Supplementary Table S8. List of peak alignments obtained by LC-MS analysis of hydrophobic extracts that showed significantly higher mean ion intensity in the TN line than in the NN line.

Ion peak ID	RT[a]	*m/z*[b]	Lipid class[c]	Acyl chain[d]	Adduct[e]	m-Score[f]	Grade[g]	Mean ion intensity (NN)	SD (NN)	Mean ion intensity (TN)	SD (TN)	Mean ion intensity ratio (TN/NN)	p-value	q-value
337	28.46	700.492	PE	18:0_13:0	$[M+Na]^+$	39.36	B	2435	4834	24172	15117	9.9	8.2.E-04	4.9.E-02
455	31.41	937.657	Hex2Cer	20:1;O3/16:1;O	$[M+NH_4]^+$	6.17	C	2205	6298	24094	13293	10.9	3.9.E-04	4.9.E-02
591	34.19	570.510	DG	10:0_21:1	$[M+NH_4]^+$	9.75	C	21835	10339	57866	26015	2.7	1.4.E-03	4.9.E-02
696	38.65	617.515	DG	18:1_18:3	$[M+H]^+$	28.08	B	9347	4379	20986	7502	2.2	9.9.E-04	4.9.E-02
961	44.87	694.671	Cer	18:1;O3/26:0	$[M+H]^+$	22.11	B	2867	8602	29789	19140	10.4	1.4.E-03	4.9.E-02
1150	47.05	930.808	AcHexSiE	23:0	$[M+NH_4]^+$	47.49	A	12891	25617	60838	24983	4.7	9.9.E-04	4.9.E-02

[a]Mean retention time (min) of the analyte.
[b]Measured *m/z* of the analyte.
[c]PE, phosphatidylethanolamines; Hex2Cer, dihexosylceramides; DG, diacylglycerides; Cer, ceramides; AcHexSiE, acyl hexosyl sitosterol esters.
[d]In PE, DG, and AcHexSiE, acyl chains were annotated as "C-atoms:double bond equivalents", and two acyl chains were separated by a separator "_" which means that the *sn*-position of acyl constituents is not known. In Cex2Cer and Cer, the sphingoid backborn was annotated as "C-atoms:double bond equivalents;O-atoms", and separated by a slash "/" from the *N*-linked fatty acid annotated as "C-atoms:double bond equivalents" or "C-atoms:double bond equivalents;O-atoms".
[e]Adduct ion of the analyte estimated by considering the mass differences between the ion of interest and all other ions in the corresponding mass spectrum.
[f]A match between the analyte and a Lipid Search database record was calculated by considering the number of product ions that matched. A higher m-Score indicates a more reliable annotation result.
[g]Degree of the structural estimation of the analyte. “Grade A” shows that both the lipid class and fatty acid chain(s) of the analyte are fully identified. “Grade B” shows that the tandem mass spectrum of the analyte contains both the lipid class-specific product ion and some fatty acid-derived product ions. “Grade C” shows that the tandem mass spectrum of the analyte contains either the lipid class-specific product ion or some fatty acid-derived product ions.

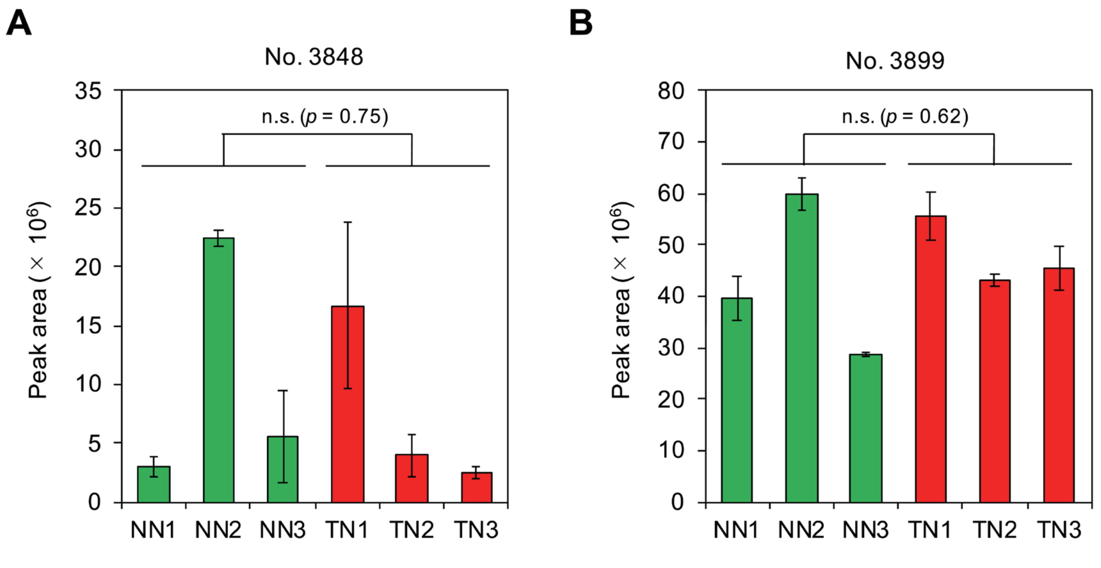

Supplementary Fig. S1. Comparison of the peak areas of two ion peaks annotated as potato steroid glycoalkaloids. Shown are results for (A) α-solanine and (B) α-chaconine sourced from the NN and TN lines.
A: The m/z 706.453 ion peak detected at retention time 16.10 min (No. 3848) was annotated as a compound with the formula C45H73NO15 (corresponding to α-solanine). B: The m/z 852.511 ion peak detected at retention time 16.19 min (No. 3899) was annotated as a compound with the formula C45H73NO14 (corresponding to α-chaconine). Data represents mean ± standard error of analytical triplicates. N.s.: not significant (Student’s *t*-test, NN vs. TN, two-tailed, $p < 0.05$)

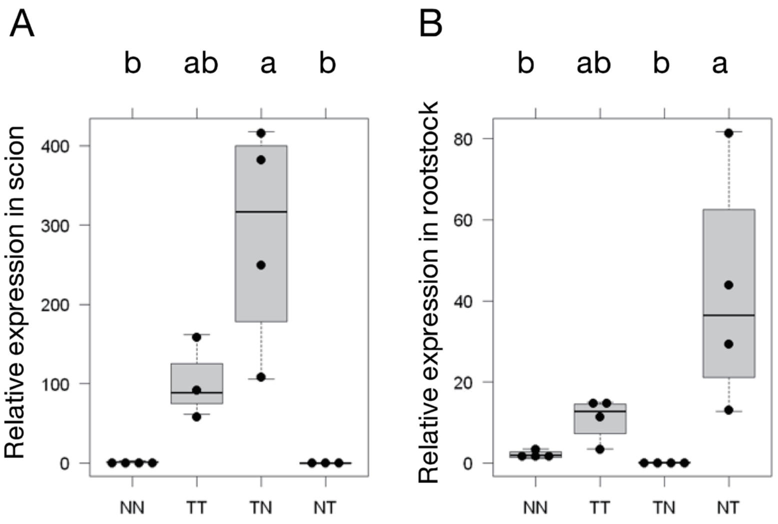

Supplementary Fig. S2. Transgene expression in transgrafted potato.
Expression levels of the *StSP6A* in the grafted potatoes were determined by quantitative RT-PCR method. Expression level of the transgenes in scions and rootstock of grafting potato plants, i.e., non-transgenic root stocks scioned with non-transgenic scions (NN), transgenic root stocks scioned with transgenic scions (TT), non-transgenic root stocks scioned with transgenic scions (TN), and transgenic root stocks scioned with non-transgenic scions (NT) cultivated under long-day condition were calculated as relative expression against expression level of endogenous ubiquitin gene and were shown A and B, respectively. Different letters above the boxes indicate significant differences among grafted plants as determined by Tukey-HSD test (α=0.05; n=6 to 8).

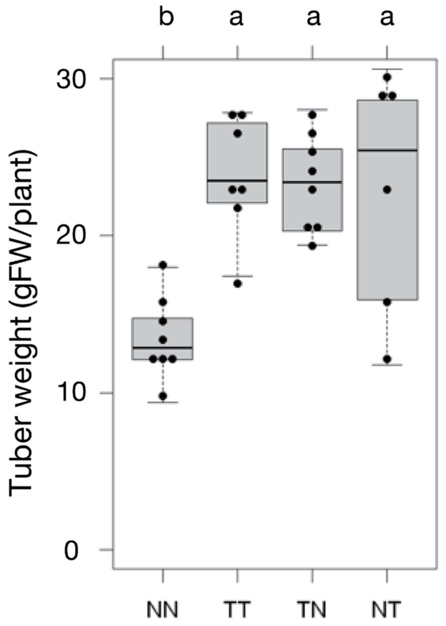

Supplementary Fig. S3. Tuber formation in long-day condition.
Grafting potato plants, i.e., non-transgenic root stocks scioned with non-transgenic scions (NN), transgenic root stocks scioned with transgenic scions (TT), non-transgenic root stocks scioned with transgenic scions (TN), and transgenic root stocks scioned with non-transgenic scions (NT) were cultivated in the growth room with 25°C in temperature and long-day cycles (16L/8D). A transgenic line 35:StSP6A #7 was used. After 3-month cultivation, all tubers were harvested and weighted for each plant. Different letters above the boxes indicate significant differences among grafted plants as determined by Tukey-HSD test (α=0.05; n=6 to 8).
